# SGLT2 inhibition restrains thyroid cancer growth via G1/S phase transition arrest and apoptosis mediated by DNA damage response signaling pathways

**DOI:** 10.1186/s12935-022-02496-z

**Published:** 2022-02-11

**Authors:** Yan Wang, Longyan Yang, Lin Mao, Lijie Zhang, Yingjun Zhu, Yongsong Xu, Yanan Cheng, Rongxin Sun, Yuanyuan Zhang, Jing Ke, Dong Zhao

**Affiliations:** 1 Center for Endocrine Metabolism and Immune Diseases, Beijing Luhe Hospital, Capital Medical University, Beijing, 101149 China; 2 Beijing Key Laboratory of Diabetes Research and Care, Beijing, 101149 China

**Keywords:** SGLT2 inhibitor, Thyroid cancer, Growth, Cell cycle, DNA damage

## Abstract

**Background:**

Although the prognosis for most patients with papillary thyroid cancer (PTC) is good, the present treatment is ineffective for 5–10% patients. Several studies found sodium–glucose cotransporter 2 (SGLT2) inhibitors may inhibit the growth of tumors. However, whether SGLT2 inhibitors have therapeutic effect on thyroid cancer remains unclear.

**Materials and methods:**

The levels of SGLT2 in PTC and normal thyroid tissue were assessed by immunohistochemistry and clinical dataset analysis. Cell growth was detected by the CCK-8 and colony formation. Glucose uptake into thyroid cancer cell was evaluated by 2-DG uptake assay. Glycolysis were analyzed by Seahorse XF Extracellular Flux Analysis. RNA-seq were used to screen differentially expressed genes of cells treated with/without canagliflozin (a SGLT2 inhibitor). Furthermore, flow cytometry, western blot, and gene set enrichment analysis were employed to elucidate cell cycle, apoptosis and the underlying mechanism of the anticancer effect of canagliflozin. The effect of canagliflozin on thyroid cancer growth was further confirmed in vivo through xenograft formation assay.

**Results:**

SGLT2 inhibition attenuated the growth of thyroid cancer cells in vitro and in vivo. Canagliflozin inhibited glucose uptake, glycolysis and AKT/mTOR signaling activation, and increased AMPK activation in thyroid cancer cell. Furthermore, canagliflozin inhibited G1/S phase transition and cyclin D1, cyclin D3, cyclin E1, cyclin E2, and E2F1 expression levels in thyroid cancer cell. In addition, canagliflozin increased apoptosis of thyroid cancer cell. Further investigation revealed that canagliflozin could increase γ-H2AX expression levels and DNA damage response signaling ATM/CHK2 activation. In thyroid cancer patients, SGLT2 was increased in thyroid cancer and positively related to cyclin D3.

**Conclusions:**

SGLT2 inhibition may limit glucose uptake resulting in energetic crisis, following oxidative stress mediated DNA damage and cell cycle arrest, which resulted to the increased cell apoptosis and decreased proliferation of thyroid cancer cells, suggesting a potential use for SGLT2 inhibitors as thyroid cancer therapeutics.

**Supplementary Information:**

The online version contains supplementary material available at 10.1186/s12935-022-02496-z.

## Introduction

Thyroid cancer is the most common malignant tumor in endocrine system, and the incidence rate has been increasing in recent years [1]. However, there are some loopholes in the clinical treatment at present, whether it is surgery, conservative endocrine therapy, or iodine radiotherapy [2, 3]. Therefore, it is essential to explore new methods or drugs for the treatment of thyroid cancer. Previous studies have shown that energy metabolism reprogramming is one of the most important features of tumor, which is characterized by high glucose uptake and enhanced glycolysis, even under sufficient oxygen condition [4–6]. High rate of glycolysis is mainly related to increased cellular glucose uptake in cancer cells. More and more evidence showed that abnormal glucose metabolism is closely related to the occurrence and development of thyroid cancer [7, 8]. Moreover, thyroid cancer cells with higher malignancy also featured with stronger glycolytic activity [9]. Therefore, targeting inhibition of glycolytic metabolism of thyroid cancer cell may be a new method for the treatment of thyroid cancer.

Sodium-glucose co-transporters 2 (SGLT2) inhibitors are a new class of oral drugs for the treatment of type 2 diabetes, including canagliflozin, dapagliflozin, etc. SGLTs are glucose transporters belonging to the solute carrier family 5A (SLC5A), which import glucose or other nutrients (mannose, galactose, fructose, myoinositols, urea, iodide, and short-chain fatty acids) into cell using the sodium concentration difference across the plasma membrane [10]. SGLT2 has received the most attention within the family. Study have found that SGLT2 was predominantly expressed at renal proximal convoluted tubules, and about 90% tubular glucose reabsorption was via SGLT2 [10]. SGLT2 inhibitors could suppress glucose reabsorption at renal tubular epithelial cells, and increase urinary glucose excretion, and finally reduce blood glucose and reverse the glucose toxicity [11]. Resent study have found SGLT2 was overexpressed in several cancers. Furthermore, SGLT2 inhibitors could attenuated the growth of cervical carcinoma [12], liver cancer [13] and breast cancer [14] by inhibiting glucose uptake in cancer cells. However, a meta-analysis showed the risk of bladder cancer might be increased with SGLT2 inhibitors [15]. Korfhage et al. recently demonstrated that canagliflozin aggravated adenoma development in mice [16]. However, the role of SGLT2 inhibitors in thyroid cancer remains unclear.

Hence, we conducted a study to investigate the effect of SGLT2 inhibitor on thyroid cancer. In this study, we explored the effect of SGLT2 inhibition on thyroid cancer cell growth through in vivo and in vitro experiments. Next, we investigated the effect of canagliflozin in glycolysis metabolism and AKT/mTOR and AMPK signaling. Furthermore, we explored the underlying mechanism of DNA damage/ATM/CHK2 mediated G1/S phase transition arrest, and the relationship between SGLT2 and cell cycle protein in thyroid cancer. The present study revealed the effect of SGLT2 inhibitor on thyroid cancer, and evaluated the clinical efficacy of SGLT2 inhibitor in preclinical animal model.

## Materials and methods

### Human subjects

We obtained 12 pairs of PTC and adjacent normal thyroid tissue from thyroidectomy conducted at the Luhe Hospital Capital Medical University. All these tissues were embedded in paraffin wax. In addition, 10 fine needle aspiration thyroid samples (6 cases of benign and 4 cases of malignancy) were collected for RNA-Seq [17]. The basic and pathological characteristics of PTC patients were extracted from medical records. Tumor staging was determined using the 7th edition of the American Joint Committee on Cancer Tumor-Node-Metastasis (AJCC-TNM) staging system. Thyroid tissue and cancer samples were from Center for Endocrine Metabolism and Immune Diseases, Beijing Luhe Hospital, Capital Medical University. All patients included in the protocol signed a declaration of informed consent. The research was approved by the Research Ethics Board of Luhe Hospital Capital Medical University and was carried out according to the World Medical Association Declaration of Helsinki.

In addition, mRNA expression data (RNA Seq v2) and clinical information for patients in The Cancer Genome Atlas thyroid cancer data set were downloaded from https://www.synapse.org and cBioPortal database (http://www.cbioportal.org), respectively, and used for analysis of differential mRNA expression and clinical prognosis. Moreover, the GEO dataset GSE3467, which consisted of 8 paired thyroid cancer and adjacent thyroid tissues, was used for analysis of differential mRNA expression.

### Cell culture and transfection

Papillary thyroid cancer cell lines TPC-1 and BCPAP cells were purchased from the National Infrastructure of Cell Line Resource (Beijing, China). Nthy-ori-3-1 cells was kindly provided by Professor. Yang Yan. TPC-1, BCPAP, and Nthy-ori-3-1 cells were cultured in Roswell Park Memorial Institute (RPMI) 1640 medium (Gibco, Cleveland, TN, USA), with 10% fetal bovine serum (FBS) (Gibco, Cleveland, TN) and 1% penicillin/streptomycin in a 37 °C/5% CO2 incubator. Lipofectamine 2000 (Invitrogen, USA) was used to transfect small interfering RNA into TPC-1 cells. Forty-eight hours after transfection, the cells were collected and analyzed by western blot. The small interfering RNA was synthetized in Sangon Biotech (Shanghai, China). The small interfering RNA sequences were: siNC:UUC UCC GAA CGU GUC ACG UTT; siSGLT2 1#:CGACAAAUACCUGGGAGCAAUTT; si SGLT2 2#:ACCAUGAUUUACACGGUGACATT.

### Proliferation assay

A Cell Counting Kit-8 (CCK8, Dojindo, Kumamoto, Japan) assay was used to assess cell proliferation rate. Cells were seeded at a density of 2000 cells/well into 96-well plates. The cells attached to the plates after 4 h incubation and were considered as 0 time point. The viable cells assessed by CCK8 assay using an Enspire microplate reader (Perkin Elmer, Waltham, MA, USA) at 450 nm.

### Colony formation assay

Cells were digested into a single cell suspension and seeded in 6-well plates (800 cells per well). After incubation for 14 days, cells were stained with crystal viole and photographed.

### Cell cycle

Cell cycles were examined by flow cytometry (FACScanto II, BD Biosciences, San Jose, CA, USA). Cells were fixed for overnight in 70% ethanol at 4 °C, and then incubated with propidium iodide and RNAase for 30 min at 37 °C before flow cytometry. ModFit software was used to analyze the data.

### Glucose uptake assay

The glucose uptake rate was evaluated using the Glucose Uptake Assay Kit (ab136955). The cells were seeded into 96-well plates at a density of 3000 cells/well. After 12 h, the cells were cultured with 10 μM canagliflozin or DMSO in completed 1640 medium for 24 h. Cells were washed with PBS and starved in 1640 medium for 12 h. Cells were starved for glucose by pre-incubating them with 100 μL KRPH buffer containing 2% BSA for 40 min, and then 2- Deoxyglucose (2-DG; 10 mmol/L) was added and cultured for 20 min. 2-DG was omitted in respective negative controls. The rest of the protocol was performed according to the instructions from the manufacturer and subjected to the measurement of the 2-DG uptake using a microplate reader at 412 nm.

### Seahorse XF Extracellular Flux analysis

The Seahorse Extracellular Flux Analyzer XF96 (Seahorse Bioscience, North Billerica, MA, USA) was used to measure the in vitro cells extracellular acidification rate (EACR) based on the manufacturer’s instructions. Briefly, 1.5 × 10^4^cells were seeded per well in the XF96-well cell culture plate and incubated at 37℃ overnight. Next day, medium was changed to bio-carbonate free DMEM with 1 mM glutamine and then cells were incubated at 37℃ for 60 min in the CO_2_ free incubator to balance the media pH and temperature. The ECAR were monitored in baseline conditions and treated with 10 mM glucose, 1 µM oligomycin, 50 mM 2-deoxy glucose (2-DG). Data were normalized by the protein quantification.

### Cell apoptosis

The apoptosis rate was evaluated by using the AnnexinV-FITC/PI Apoptosis Detection kit according to the instructions from the manufacturer. The cells were seeded into 6-well plates. Following starvation for 24 h (serum-free medium), the cells were collected, washed with PBS, and resuspended in 500μL Binding buffer. Then, 5μL Annexin V-FITC and 5μL PI were added to the buffer and incubated at room temperature for 15 min in the dark. Cells were analyzed by flow cytometry within 1 h. Annexin V positive cells were considered to be apoptotic cells.

### RNA sequencing

Briefly, BCPAP cells were treated with 10 μM of canagliflozin or DMSO as negative control with three biological replicates for each group. After incubation for 36 h, cells were collected and total RNA was extracted using the TRIzol Reagent according to the manufacturer's instructions (Invitrogen). RNA quality was determined by 2100 Bioanalyser (Agilent) and quantified using the ND-2000 (NanoDrop Technologies). Then RNA prepared for library preparation and sequencing using the Illumina Hiseq2000 platform of Majorbio Biotech (Shanghai, China). The data were analyzed on the free online Majorbio Cloud Platform (www.i-sanger.com) according to the instructions.

### Detection of reactive oxygen species (ROS)

Cells (5 × 10^5^/well) were seeded in 6-well plates. After culturing overnight, cells were cultured with 10 μM canagliflozin or DMSO in medium for 24 h. Cells were then washed and re-suspended in PBS containing 10 μM of DCFH-DA and kept at 37 °C for 30 min in the dark. Next, cells were washed and analyzed by flow cytometry. Data processing was performed using FlowJo software version 10.5.0 for Windows (FlowJo LCC, Ashland, OR, USA).

### In vivo xenograft formation assay

This study was performed following the Guide for the Care and Use of Laboratory Animals by National Institutes of Health, and all procedures were approved by the Animal Care and Use Committee of Capital Medical University.

TPC-1 cells were subcutaneously implanted in each of 5-week-old male Balb/c nude mice (1 × 10^5^ cells in 0.1 ml PBS). Mice were then randomly divided into two groups when tumor volume grew to 80–100 mm^3^: vehicle control (0.5% CMC + 0.25% Tween 80) and canagliflozin group. The mice were monitored every two days for the growth of tumors, and they were sacrificed after 4 weeks. For euthanization, the mice were intraperitoneally injected with 100 mg/kg of sodium pentobarbital. The tumor xenografts were dissected and weighted after the deaths of the mice. Tumor volumes were estimated according to the equation: volume = width (mm) × width (mm) × length (mm)/2.

### Immunohistochemistry (IHC)

The samples used for immunohistochemistry analysis include human tissues and mice tumor xenograft. Immunohistochemistry was performed as described previously [18]. Primary antibodies were incubated at the optimal conditions (SGLT2, 1:100, Abcam; Ki67, 1:100, Santa Cruz). Histochemistry score (H-SCORE) for thyroid cancer tissue and adjacent tissue were recorded separately to measure the expression levels of SGLT2. The staining intensity was transformed into corresponding values (0, negative; 1 + , weak; 2 + , moderate; and 3 + , strong). Based on positive cell number and staining intensity value, H-score was calculated as the following formula: H-SCORE = ∑(PI × I) = (percentage of cells with weak intensity × 1) + (percentage of cells with moderate intensity × 2) + (percentage of cells with strong intensity × 3).

### Gene set enrichment analysis

The gene sets were obtained from the Molecular Signatures Database of the Broad Institute (http://software.broadinstitute.org/gsea/msigdb). Tests were performed by using default settings, with permutation number set at 1000. A false discovery rate (FDR) of < 0.25 was considered to indicate a statistically significant difference.

### Cell migration and invasion assay

See Additional file 1: Wound-healing and transwell invasion assay.

### Statistical analysis

Statistical analysis was performed using SPSS 18.0 (SPSS Inc., Chicago, IL, USA). Results are expressed as mean ± SD. Two-tailed unpaired Student’s t-test and repeated-measures analysis of variance were used to determine statistical significance. Statistical significance was accepted for p < 0.05.

## Results

### SGLT2 inhibition suppressed thyroid cancer cells growth

**Fig. 1:**
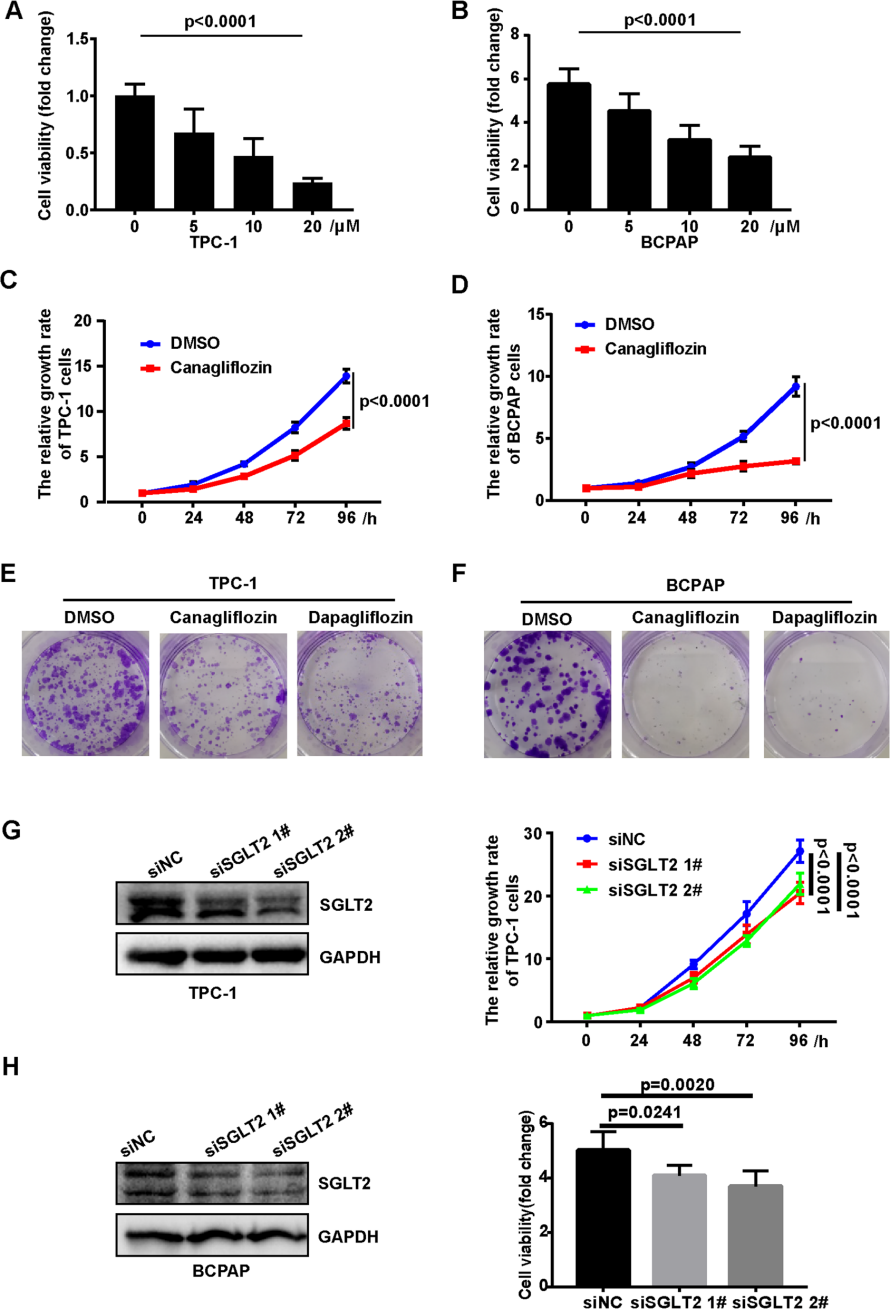
The effect of SGLT2 inhibition on growth of TPC-1 and BCPAP cells. **A**, **B** Canagliflozin inhibited the viability of TPC-1 and BCPAP cells. TPC-1 and BCPAP cells were treated with 0, 5, 10, 20 μM canagliflozin for 48 h, then cell viability were measured by CCK8. One-way ANOVA were used to determine statistical significances, p < 0.0001. **C**, **D** Canagliflozin inhibited TPC-1 and BCPAP cells proliferation. TPC-1 and BCPAP cells were treated with 10 μM canagliflozin, then viable cells were measured at 0, 24, 48, 72, 96 h by CCK8. Repeated-measures analysis of variance were used to determine statistical significances, p < 0.0001. **E**, **F** Canagliflozin inhibited TPC-1 and BCPAP cells colony formation. TPC-1 and BCPAP cells were treated with 10 μM canagliflozin or dapagliflozin for 14 days, then colony formation was monitored by crystal violet stain. **G** Knockdown of SGLT2 inhibited the proliferation of TPC-1 cells. 24 h after transfection, the cells were plated into 96-well plates, and measured at 0, 24, 48, 72, 96 h by CCK8. Knockdown of SGLT2 in TPC-1 cells was verified by Western blot analysis. **H** Knockdown of SGLT2 inhibited cell viability of BCPAP cells. 24 h after transfection, cells were cultured in 96-well plates and stained with CCK8 at 72 h. Knockdown of SGLT2 in BCPAP cells was verified by western blot analysis

To explore the effect of SGLT2 inhibitor on thyroid cancer cells, TPC-1 and BCPAP were used to evaluated the effect of canagliflozin on cell growth. As shown, canagliflozin attenuated the proliferation of TPC-1 and BCPAP cells in a dose and time-dependent manner (Fig. 1A, B and Fig. 1C, D). Furthermore, colony formation was measured, and the data showed that canagliflozin decreased clonogenicity of TPC-1 and BCPAP (Fig. 1E, F). Another SGLT2 inhibitor-dapagliflozin showed similar effect with canagliflozin on the proliferation of TPC-1 and BCPAP cells (Fig. 1E, F and Additional file 2: Fig. S1). To further confirmed the effect of SGLT2 inhibition on cell proliferation, SGLT2 was knocked-down in TPC-1 and BCPAP cells. The results showed that knockdown of SGLT2 inhibited the proliferation of TPC-1 and BCPAP (Fig. 1G, H). Putting together, these data demonstrated SGLT2 inhibition suppressed the growth of thyroid cancer cell.

Additionally, the cytotoxicity of SGLT2 inhibitor on normal thyroid cell were detected. The proliferation and colony formation of Nthy-ori-3-1 cell were performed. The results showed that there was no significant difference in the proliferation and colony formation of Nthy-ori-3-1 cells treated with and without canagliflozin (Additional file 3: Fig. S2). Dapagliflozin showed the similar results (Additional file 3: Fig. S2). These results suggested that SGLT2 inhibitor had no effect on the growth of normal thyroid cell.

**Fig. 2:**
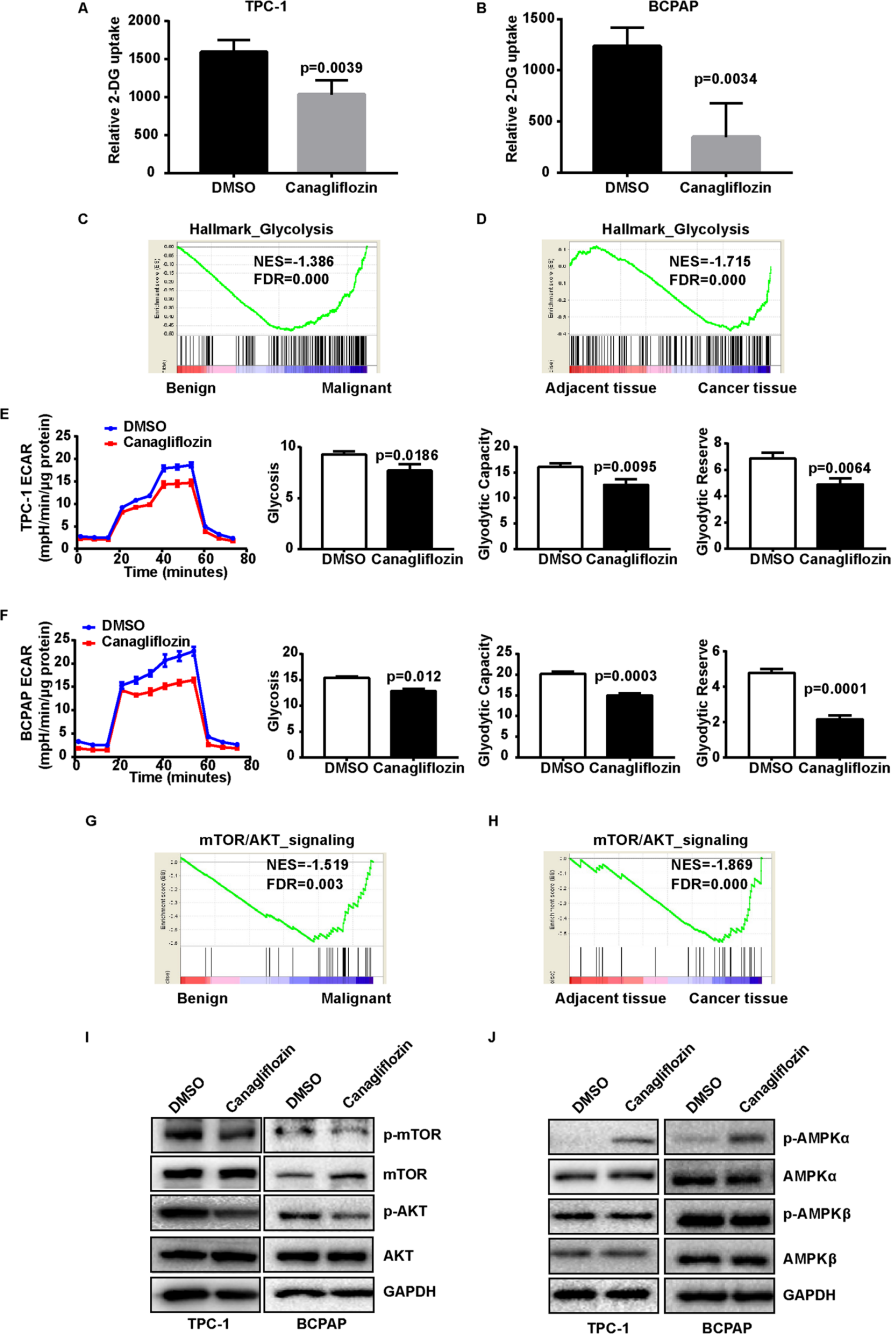
The effect of canagliflozin on glucose uptake and glycolysis of TPC-1 and BCPAP cells. **A**, **B** Canagliflozin inhibited 2-DG uptake in TPC-1 and BCPAP cells TPC-1 and BCPAP cells were treated with 10 μM canagliflozin for 24 h, and 2-DG uptake were measured by Glucose Uptake Assay Kit. A t-TEST was used to determine statistical significance. **C** Gene signatures for glycolysis were enriched in malignant thyroid cancer in patients. **D** Gene signatures for glycolysis were enriched in thyroid cancer tissue in TCGA dataset. **E**, **F** Canagliflozin inhibited glycolysis level in TPC-1 and BCPAP cells. TPC-1 and BCPAP cells were treated with 10 μM canagliflozin for 24 h, then the ECAR were monitored in baseline conditions and treated with 10 mM glucose, 1 µM oligomycin, and 50 mM 2- DG. Cells were collected in 100 μL lysis, and detected protein concentration by BCA kit. The date were analyzied by Seahorse XF-Glycolysis Stress Test. The ECAR were normalized by the protein quantification. **G** Gene signatures for AKT/mTOR activation were enriched in malignant thyroid cancer in patients. **H** Gene signatures for AKT/mTOR activation were enriched in thyroid cancer tissue in TCGA dataset. **I**, **J** Canagliflozin reduced the phosphorylation of AKT and mTOR and increased the phosphorylation of AMPK. Cells were treated with 10 μM canagliflozin for 24 h, then the cells were collected, p-AKT, AKT, p-mTOR, mTOR, p-AMPKα, AMPKα, p-AMPKβ, and AMPKβ were detected by western blot. GAPDH was used as a loading control

The effect of canagliflozin on migration and invasion were detected by wounding healing assay and transwell assay, and the result showed that canagliflozin had no effect on migration of thyroid cancer cells, but inhibited invasion of thyroid cancer cell (Additional file 4: Fig. S3).

**Fig. 3:**
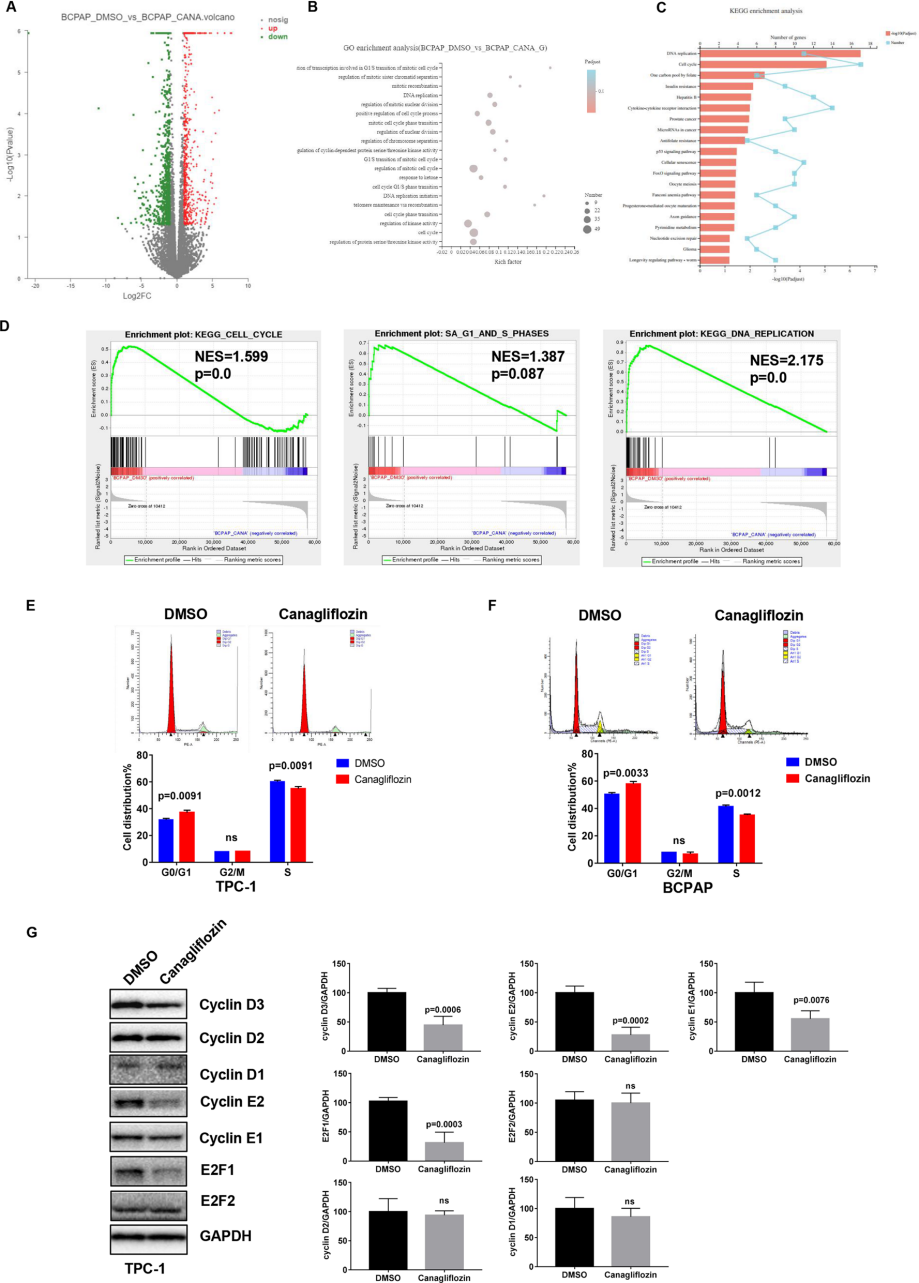
RNA-seq analysis and the effect of canagliflozin on cell cycle in thyroid cancer. **A**–**D** RNA sequencing results: differential gene expression; GO term analysis; KEGG pathway analysis; GSEA. **E**, **F** Canagliflozin inhibited G1/S phase transition of TPC-1 and BCPAP cells. Cells were treated with 10 μM canagliflozin for 24 h, then the cell cycle was detected by flow cytometry. **G** Canagliflozin inhibited G1/S phase transition related protein expression. Cells were treated with 10 μM canagliflozin for 24 h, then cyclin D1-3, cyclin E1-2, E2F1-2 were detected by western blot. GAPDH was used as a loading control

### Canagliflozin interfered glucose uptake and glycolysis in thyroid cancer cell

It was reported that canagliflozin could decrease the blood glucose level by blocking glucose reabsorption in renal tubular epithelial cells. To investigate whether SGLT2 inhibitor could suppress glucose absorption in thyroid cancer cells as renal tubular epithelial cell, 2-DG uptake assay was performed in TPC-1 and BCPAP by using canagliflozin. The results showed that cells treated with canagliflozin had lower 2-DG uptake rates comparing with control (Fig. 2A, B). These results confirmed that SGLT2 inhibitor could suppress the glucose uptake into thyroid cancer cell. Previous studies have shown that energy metabolism reprogramming was one of the important characteristics of cancer, and cancer cells largely produced energy via high-rate glycolysis. The results of gene set enrichment analysis (GSEA) on the Malignant/Benign nodules RNA-seq data showed that genes related to glycolysis were enriched in malignant tumors compared with benign thyroid nodules (Fig. 2C). Similarly, the results of GSEA on TCGA data also showed that genes related to glycolysis were enriched in thyroid cancer tissue compared with adjacent tissue (Fig. 2D). These results indicated that glycolysis was overactivited in thyroid cancer. Therefore, we analyzed the effect of SGLT2 inhibitor on glycolysis of thyroid cancer cells by using Seahorse XF Extracellular Flux assay. The results showed that cells treated with canagliflozin had lower EACR, glyodytic capacity, and glyodytic reserve comparing with control. (Fig. 2E, F). These results confirmed that SGLT2 inhibitor could suppress the glycolysis level of thyroid cancer cell. Putting together, SGLT2 inhibitor interfered glucose uptake and glycolysis in thyroid cancer cell.

### Canagliflozin inhibited AKT/mTOR pathway and promoted AMPK pathway activation in thyroid cancer cell

AKT/mTOR pathway and AMPK pathway has been proved to be related to cancer progress and cell energy metabolism. The results of GSEA on the Malignant/Benign nodules RNA-seq data showed that genes related to AKT/mTOR signaling were enriched in malignant tumors compared with benign thyroid nodules (Fig. 2G). Similarly, the results of GSEA on TCGA data also showed that genes related to AKT/mTOR signaling were also enriched in thyroid cancer tissue compared with adjacent tissue (Fig. 2H). These results indicated that AKT/mTOR signaling was overactivated in thyroid cancer. Then we investigated whether AKT/mTOR pathway and AMPK pathway were involved in the effects of canagliflozin on thyroid cancer growth. As shown, canagliflozin decreased the phosphorylation of AKT and mTOR in TPC-1 and BCPAP cell (Fig. 2I). At the same time, canagliflozin increased the phosphorylation of AMPKα, not AMPKβ, in TPC-1 and BCPAP cell (Fig. 2J). Taken together, canagliflozin inhibited AKT/mTOR pathway and promoted AMPK pathway activation in thyroid cancer cell.

### Canagliflozin induced cell cycle arrest at G1/S checkpoint in thyroid cancer cell

In order to further explore the mechanism behind canagliflozin-inhibited the growth of thyroid cancer cell, we conducted RNA-sequencing. BCPAP cells were treated with 10 μM canagliflozin and DMSO as negative control. Cells were cultured for 36 h and sent for RNA sequencing. Differential genes expression were presented as a scatter plot (Fig. 3A). Using GO term analysis, we found that the differential genes expression played important roles in regulating cell cycle and DNA replication, especially G1/S phase transition (Fig. 3B). Meanwhile, GSEA and KEGG pathway analysis showed that most deferentially expressed genes were enriched in cell cycle, G1/S phase transition, and DNA replication (Fig. 3C, D). To validate the above RNA-seq results, cell cycle progression was detected by flow cytometry, and the results showed after treatment with canagliflozin, the percentage of TPC-1 in G0/G1 phase increased from 31.76% to 37.12% (p = 0.0091), whereas the distribution of cells in S phase decreased from 60.24% to 54.88% (p = 0.0091) (Fig. 3E). Consistent results were received in BCPAP (Fig. 3F). To further confirm the effect of canagliflozin on G1/S phase transition, G1/S phase cell cycle-related proteins were investigated by western blot. As shown in Fig. 3G, canagliflozin inhibited the expression levels of cyclin D1, cyclin D3, cyclin E1, cyclin E2, and E2F1. These data demonstrated that canagliflozin induced cell cycle arrest at G1/S checkpoint in thyroid cancer cell.

### SGLT2 inhibition induced DNA damage and ATM/CHK2 pathway activation in thyroid cancer

**Fig. 4:**
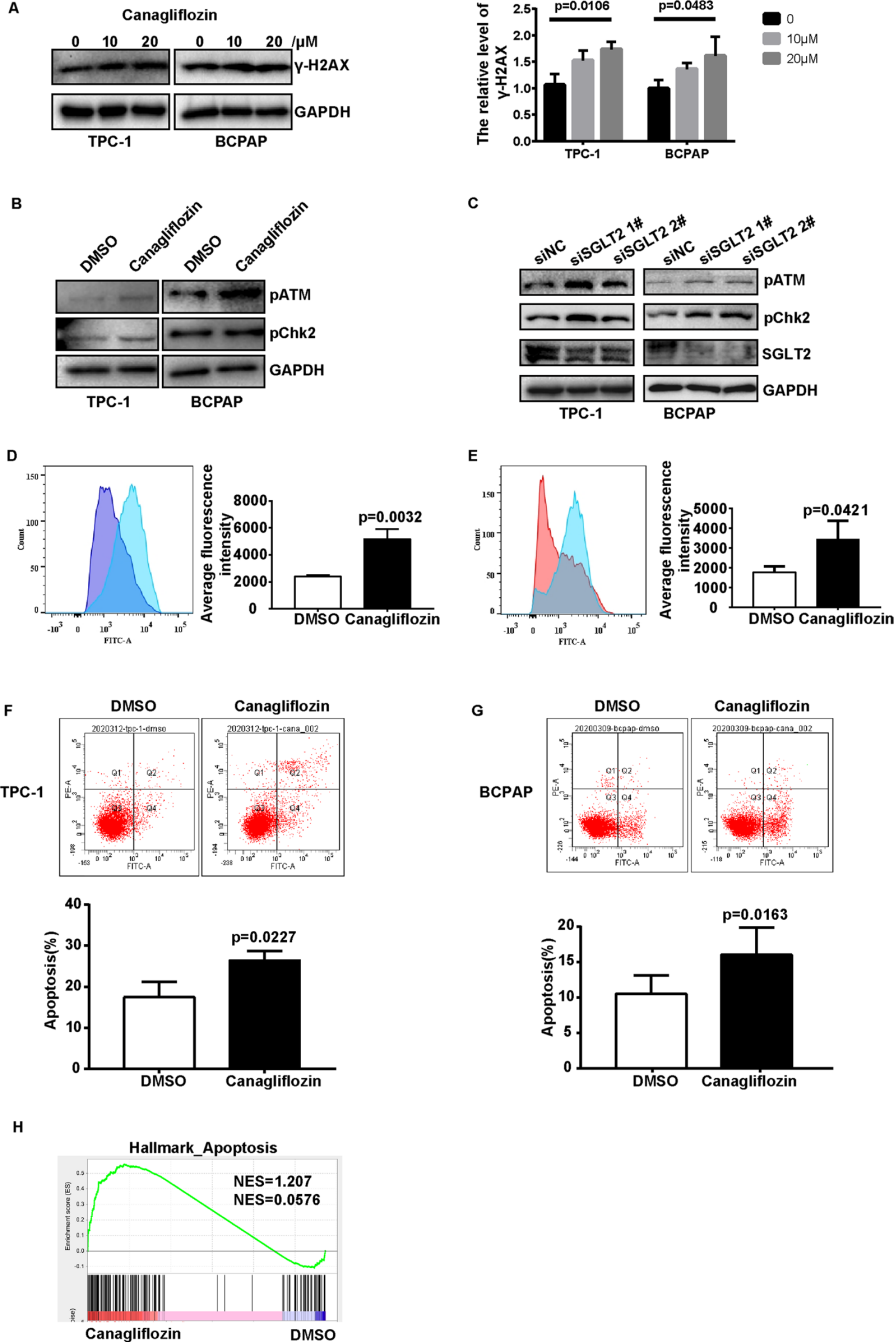
The effect of canagliflozin on DNA damage response and apoptosis in thyroid cancer. **A** Canagliflozin induced γ-H2AX expression in TPC-1 and BCPAP cells. TPC-1 and BCPAP cells were treated with 0, 10, 20 μM canagliflozin for 24 h, and γ-H2AX expression levels were detected by western blot. GAPDH was used as a loading control. One-way ANOVA were used to determine statistical significances. **B** Canagliflozin increased the phosphorylation of ATM and CHK2 in TPC-1 and BCPAP cells. TPC-1 and BCPAP cells were treated with 10 μM canagliflozin for 24 h, and the p-ATM and p-CHK2 were detected by western blot. GAPDH was used as a loading control. **C** Knockdown of SGLT2 increased p-ATM and p-CHK2 expression levels both in TPC-1 and BCPAP cells. 48 h after transfection, the cells were collected and analyzed by western blot. GAPDH was used as a loading control. **D**, **E** Canagliflozin induced ROS accumulation in TPC-1 and BCPAP cells. TPC-1 and BCPAP cells were treated 10 μM canagliflozin for 24 h, and cells were stained with DCFH-DA following flow cytometer analysis. A t-TEST was used to determine statistical significances. **F**, **G** Canagliflozin promoted cell apoptosis of TPC-1 and BCPAP cells. TPC-1 and BCPAP cells were treated 10 μM canagliflozin for 24 h, and cells were stained with Annexin V and PI following flow cytometer analysis. **H** Gene signatures for cell apoptosis were enriched in a subgroup treated with canagliflozin

DNA damage and DNA damage response signaling-ATM/CHK2 pathway played important roles in regulation of G1/S phase transition in cancer cell. To investigate the effects of canagliflozin on DNA damage in thyroid cancer cells, γ-H2AX levels were investigated by western blot. The results showed that canagliflozin could induced the expression of γ-H2AX in TPC-1 and BCPAP cells (Fig. 4A). Then, the effect of canagliflozin on the regulation of DNA damage response signaling were analyzed, and found that the phosphorylation of ATM and CHK2 were increased in canagliflozin treatment group comparing with control group in TPC-1 and BCPAP cells (Fig. 4B). To further confirmed the effect of SGLT2 inhibition on ATM/CHK2 pathway, SGLT2 was knocked-down in TPC-1 and BCPAP cells. The results showed that knockdown of SGLT2 enhanced the activation of ATM and CHK2 in TPC-1 and BCPAP (Fig. 4C). Excessive production of reactive oxygen species (ROS) in cells could lead to DNA damage. Then, the levels of ROS were detected, and the results showed the ROS levels were increased upon canagliflozin treatment in TPC-1 and BCPAP cells (Fig. 4D, E). These results suggested that SGLT2 inhibition induced ROS accumulation, DNA damage, and ATM/CHK2 pathway activation in thyroid cancer.

### Canagliflozin induced cell apoptosis of thyroid cancer

The effect of canagliflozin on apoptosis were evaluated by flow cytometry and found canagliflozin enhanced the apoptosis rate of TPC-1 and BCPAP cells (Fig. 4F, G). Furthermore, the results of GSEA on BCPAP-RNA-seq data showed that genes related to cell apoptosis were enriched in canagliflozin treatment group comparing with DMSO treatment group (Fig. 4H). These results confirmed that SGLT2 inhibition induced cell apoptosis of thyroid cancer.

### Canagliflozin inhibited thyroid cancer growth in vivo

**Fig. 5:**
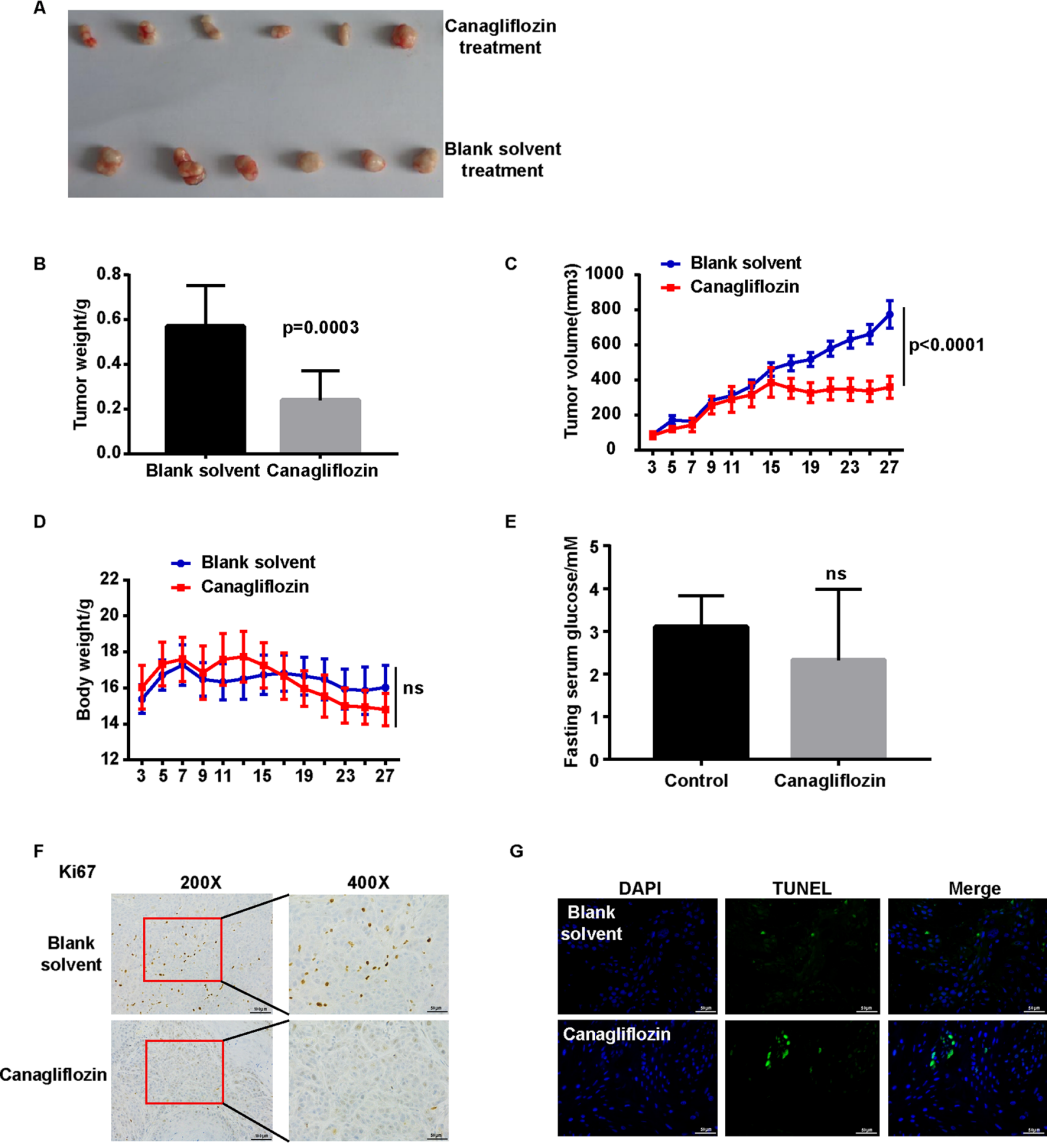
Canagliflozin inhibited thyroid cancer growth in vivo. **A** The representative image of xenografts. **B** Tumor weights of canagliflozin treatment group were smaller than those treatment with blank solvent. The xenograft tumors were dissected to detect the weights at 35 days after transplantation. A t-TEST was used to determine statistical significances. **C** The growth curve of subcutaneous xenograft tumor from TPC-1 cells in nude mice. Tumor size was measured every 2 days. Repeated-measures analysis of variance was used to determine statistical significances. **D** The body weights of mice administrated with canagliflozin or blank solvent throughout the experimental period. Repeated-measures analysis of variance was used to determine statistical significances. **E** Canagliflozin had no effect of fasting blood glucose in mice with thyroid cancer allografts. A t-TEST was used to determine statistical significances. F. Ki67 staining of thyroid carcinoma xenografts. **G** Tunel staining of thyroid carcinoma xenografts

A tumor xenograft mouse model was used to assess the anti-cancer activity of canagliflozin in vivo. Tumor-bearing mice received the same volume 0.5% CMC + 0.25% Tween 80 (blank solvent) or canagliflozin (100 mg/kg.bw) for 28 days by the oral administration. As shown in Fig. 5A, canagliflozin suppressed the growth of thyroid cancer xenografts. Accordingly, the weight and volume of tumors were decreased in canagliflozin treatment group as compared with the control (Fig. 5B, C). Furthermore, canagliflozin had no effect on the body weights and fasting blood sugar of thyroid cancer cell xenograft mouse (Fig. 5D, E). Immunohistochemical staining analysis showed that canagliflozin decreased the expression of Ki67, a cell proliferation marker (Fig. 5F). Meanwhile, more TUNEL positive cells were observed in canagliflozin treatment tumor comparing with control tumor (Fig. 5G). These results confirmed that canagliflozin inhibited thyroid cancer growth in vivo.

### SGLT2 levels were increased in thyroid cancer

**Fig. 6:**
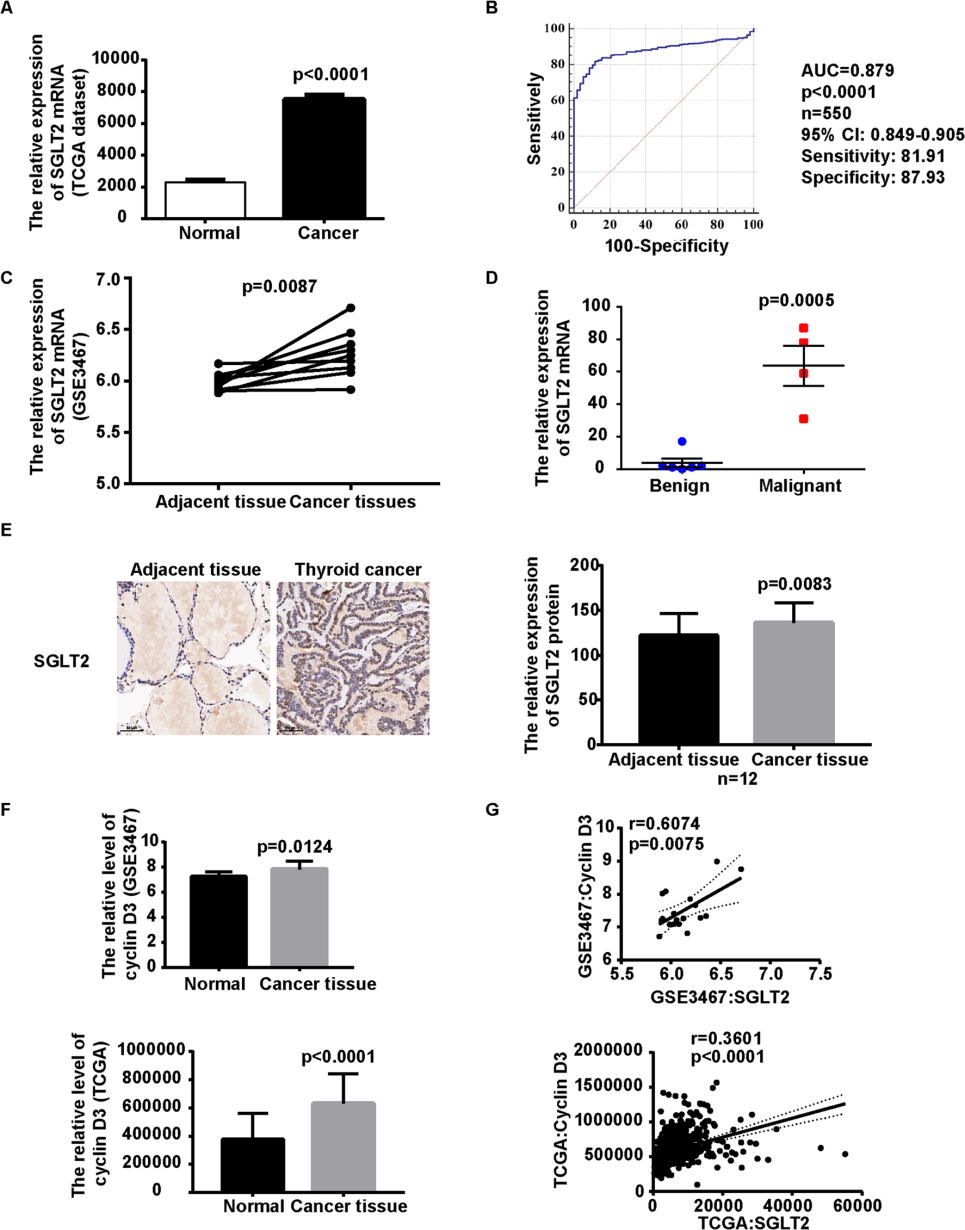
SGLT2 was increased and positively related to cyclin D3 in thyroid cancer patients. **A** SGLT2 levels were increased in thyroid cancer comparing with adjacent tissue in TCGA dataset. **B** Receiver operating characteristic curve for SGLT2 as a diagnostic criterion for thyroid cancer. **C** SGLT2 levels were increased in thyroid cancer comparing with paired adjacent tissue in GEO dataset. **D** SGLT2 levels were increased in thyroid cancer comparing with benign thyroid nodules. **E** SGLT2 protein level was increased in thyroid cancer as comparing with adjacent tissue detected by IHC. Representative images of SGLT2 IHC staining in thyroid cancer and its adjacent tissue (Left panel). Paired t-TEST was used for comparison. **F** The levels of cyclin D3 were increased in thyroid cancer tissue in GEO and TCGA dataset. **G** The levels of SGLT2 were positively related with cyclin D3 in GEO and TCGA dataset. A t-TEST was used to determine statistical significances

The levels of SGLT2, the target of canagliflozin, were analyzed using several clinical thyroid cancer dataset. First, we found the levels of SGLT2 were higher in thyroid cancer tissue comparing with adjacent tissue or paired adjacent tissue in TCGA dataset (Fig. 6A). Meanwhile, the SGLT2 levels were a potential biomarker for patients with thyroid cancer in TCGA dataset, with an AUC of 0.879 (95% confidence interval: 0.849‑0.905), a sensitivity value of 81.91% and a specificity value of 87.93% (Fig. 6B). Next, the result was further confirmed in thyroid cancer GEO dataset (GSE3467) (Fig. 6C). Then, SGLT2 mRNA levels were analyzed in RNA-seq dataset of fine needle aspiration thyroid samples (6 cases of benign thyroid node and 4 cases of malignant PTC). Compared with benign thyroid nodules, the SGLT2 mRNA levels were increased in thyroid cancer (Fig. 6D). Furthermore, 12 pairs of PTC tissues and adjacent normal thyroid tissues were collected based on postoperative pathological reports. The clinicopathologic characteristics of patients with PTC are shown in Table 1. IHC staining for SGLT2 were displayed in Fig. 6E, and the levels of SGLT2 were quantified according to the IHC scoring systemthe. The result suggested that the protein levels of SGLT2 were increased in thyroid cancer as comparing with adjacent tissue (Fig. 6E). Taking together, these data suggested that SGLT2 levels were increased in thyroid cancer.

**Table1:** Characteristics of patients with PTC

Variable	No
No. of patients	12
Age (mean ± SD) (in years)	41.42 ± 12.24
Gender	
Male	1
Female	11
Size(cm)	
≦ 5	9
> 5	3
T Stage	
T1	9
> T1	3
Lymph node metastasis	
N0	4
N1	8
Stage	
I	4
> I	8

In addition, Cyclin D3 played an important role in regulation of cancer cell growth and glycolysis, and we found the levels of cyclin D3 were increased in thyroid cancer tissue, and positively related with the levels SGLT2 in GEO and TCGA dataset (Fig. 6F, G). These results suggested that SGLT2 levels were increased in thyroid cancer and positively related with cyclin D3.

## Discussion

More and more evidences showed that patients with diabetes had a higher cancer risk and mortality rate [19]. Glucose plays a critical role in metabolism of many tumor types. Cancer metabolism is characterized by high rate of glycolysis and glucose uptake, which maintains cancer cell growth. In our present study, GSEA results futher confirmed glycolysis was overactivated in thyroid cancer (Fig. 2C, D). Recent studies have highlighted the effects of several antidiabetic drugs on thyroid cancner. Kebebew groups found that metformin could inhibit the glucose uptake and inhibit the proliferation, migration and epithelial mesenchymal transition of thyroid cancer cells, and promote the apoptosis of cancer cells [20, 21]. Previous study have found SGLT2 inhibitors attenuated cervical carcinoma [12], liver cancer [13] and breast cancer [14] growth. The effect of SGLT2 inhibitor on thyroid cancer remains unknown. Our results showed SGLT2 expression was increased in thyroid cancer comparing with thyriod tissue (Fig. 6), and SGLT2 inhibitors could inhibit growth of thyroid cancer cell (Fig. 1 and Additional file 2: Fig. S1).

High rate of glucose uptake and glycolysis provides a large amount of adenosine triphosphate (ATP) for the growth of tumor cells, and creates a suitable microenvironment for tumor cells to survive [22]. Our results suggested SGLT2 could be act as a glucose transporter in thyroid cancer cell, and SGLT2 inhition could suppress glucose uptake and glycolysis level (Fig. 2E, F). AKT/mTOR pathway and AMPK pathway have been proved to be related to thyroid cancer progress and cell energy metabolism [23]. Soravis Osataphan et al. demonstrated that canagliflozin reprogramed systemic metabolism via AMPK/mTOR signaling [24]. Here, we confirmed that canagliflozin inhibited the activation of AKT/mTOR pathway, and promoted AMPK signaling activation in thyroid cancer cell. However, it needs to be further studied how canagliflozin regulates these signaling pathways, and whether the changes of AKT/mTOR and AMPK pathway are the result or the reason for inhibition of glycolysis.

In our study, the GO, KEGG and GSEA analysis of RNA-seq of BCPAP cell treatment with canagliflozin showed SGLT2 inhibition had a strong influence on G1/S phase transition. Cell cycle assay and the analysis of G1/S phase transition related protein levels further confirmed the results (Fig. 3). Previous study have found canagliflozin could inhibited cyclin A in HUVECs [25], or induced G2/M arrest in hepatocellular carcinoma [13], or induced G1/G0 phase arrest in breast cancer [14]. The different effect of canagliflozin on cell cycle may due to tissue specificity and different drug concentration. Interestingly, our result revealed the levels of SGLT2 were positively related with cyclin D3 in thyroid cancer patients (Fig. 6). Cyclin D3 played a crucial role in mTOR-midiated cell cycle regulation. Alexandra et al. demonstrated forskolin-mediated cAMP-dependent protein kinase A stimulation induced mitogenesis that was dependent upon mTOR and specifically increased the level and activation of cyclin D3 in 3T3 cells [26]. However, the regulationship of SGLT2 and cyclin D3 needs further study in the future.

SGLT2 inhibition has been reported in several cancers. Previous study have found canagliflozin inhibited phosphorylation of ERK, p38 and AKT and cleavage of caspase3 in liver cancer [13]. SGLT2 inhibitors increased the phosphorylation of AMPK and decreased the phosphorylation of 70 kDa ribosomal protein S6 kinase 1 (p70S6K1) in breast cancer cells [14]. SGLT2 silencing or inhibition suppressed Hippo signaling activation in pancreatic cancer [27]. Empagliflozin activated the AMPK/FOXA1 pathway and inhibited the expression of Sonic Hedgehog Signaling Molecule in cervical cancer [28]. However, the mechanisms underlying the effects of SGLT2 inhibitor on thyroid cancer remains unclear. It is important to maintain the integrity of genomic DNA for the growth of cancer cells [29, 30]. Cancer cells may suffer from different degrees of DNA damage due to chemotherapeutic factors, and DNA damage response is the DNA modification initiated to protect from DNA damage, mainly including the activation of DNA damage repair, cycle checkpoint, and DNA damage induced apoptosis [31, 32]. γ-H2AX is the sensitive markers of DNA damage [33, 34]. Our result showed canagliflozin increased γ-H2AX levels in thyroid cancer. The activation of ATM/CHK2 signaling is one of the key points involved in DNA damage recognition and repair through homologous recombination and non-homologous end joining recombination, which leads to cell cycle arrest [35]. We found that canagliflozin increased the activition of ATM/CHK2 in thyroid cencer cell, indicating DNA damage repair initiated, which may be related to insufficient energy in cancer cell. As we know, once the DNA damage cannot be repaired, p53 or other pro-apoptotic factors would be activated to start the apoptotic process and clear the damaged cells36. The KEGG enrichment analysis showed p53 signaling pathway changed significiently in canagliflozin-treatment group (Fig. 3C). Furthermore, our results revealed canagliflozin could induced thyroid cancer cell apoptosis as expected. Previous study had confirmed that glucose deprivation impaired glycolysis and led to oxidative stress due to increased production of ROS and impaired antioxidant system [37]. Oxidative stress played an important roles in DNA damage and DNA damage response signaling-ATM/CHK2 pathway in cancer cell [38]. Villani et al. found canagliflozin inhibited mitochondrial complex-I to limit cancer cell proliferation [39]. Mitochondrial complex I inhibition was found to trigger ROS increase [40]. In our preasent study, we found canagliflozin induced ROS accumulation in thyroid cancer cells. Therefore, canagliflozin induced ROS-mediated DNA damage and ATM/CHK2 activation, which lead to G1/S phase transition arrest and increased apoptosis in thyroid cancer.

The present study revealed the effect of SGLT2 inhibitor on thyroid cancer, and evaluated the clinical efficacy of SGLT2 inhibitor in preclinical animal model. Several studies found diabetes was one of the risk factors of thyroid cancer, and the research results have theoretical significance for the prevention and treatment of thyroid cancer in diabetic patients.

## Conclusions

In conclusion, our study revealed canagliflozin could inhibited the thyroid cancer cell growth, suggesting a potential use for SGLT2 inhibitors as thyroid cancer therapeutics. The underlying molecular mechanism include: (1) SGLT2 inhibition inhibited glucose uptake and glycolysis level, and inhibited AKT/mTOR activation and increased AMPK activation, which lead to decreased proliferation of thyroid cancer. (2) SGLT2 inhibition induced ROS-mediated DNA damage and ATM/CHK2 activation, which lead to G1/S phase transition arrest and increased apoptosis in thyroid cancer.

## Supplementary Information

**Additional file 1:** Wound-healing and transwell invasion assay.

**Additional file 2:** **Figure S1.** Dapagliflozin inhibited TPC-1 and BCPAP cells growth. A,B. Dapagliflozin inhibited TPC-1 and BCPAP cells viability. TPC-1 and BCPAP cells were treated with 0, 20, 40, 80μM dapagliflozin for 48 h, then cell viability were measured by CCK8. One-way ANOVA were used to determine statistical significance. C,D. Dapagliflozin inhibited TPC-1 and BCPAP cells proliferation. TPC-1 and BCPAP cells were treated with 40μM dapagliflozin, then viable cells were measured at 0, 24, 48, 72, 96h by CCK8. Repeated-measures analysis of variance were used to determine statistical significance.

**Additional file 3:** **Figure S2.** SGLT2 inhibition had no effect on normal thyroid epithelial cell. A. Canagliflozin had no effect on Nthy-ori-3-1 cells proliferation. Nthy-ori-3-1 cells were treated with 10 μM canagliflozin, then viable cells were measured at 0, 24, 48, 72, 96h by CCK8. Repeated-measures analysis of variance were used to determine statistical significance, p>0.05. B. Dapagliflozin had no effect on Nthy-ori-3-1 cells proliferation. Nthy-ori-3-1 cells were treated with 20 μM dapagliflozin, then viable cells were measured at 0, 24, 48, 72, 96h by CCK8. Repeated-measures analysis of variance were used to determine statistical significance, p>0.05. C. Canagliflozin or dapagliflozin had no effect on Nthy-ori-3-1 cells colony formation. Cells were treated with 10μM canagliflozin or 40μM dapagliflozin for 14 days, then colony formation was monitored by crystal violet stain.

**Additional file 4:** **Figure S3.** The effect of SGLT2 inhibitor on thyroid cancer migration and invasion. A. Canagliflozin had no effect on TPC-1 cells migration by the wound-healing assay. B. Canagliflozin had no effect on TPC-1 and BCPAP cells invasion. The Boyden chambers invasion assay was used. We counted the numbers of cells in light microscopy fields at ×200 magnification.

## Data Availability

All data supporting the conclusions of this manuscript are provided in the text and figures. Please contact the author for data requests.

## Abbreviations

SGLT2: Sodium dependent glucose transporters 2
CCK8: Cell Counting Kit-8
IHC: Immunohistochemistry
ROS: Reactive oxygen species
GSEA: Gene set enrichment analysis
TCGA: The Cancer Genome Alters
