# Supplementary material for: SGLT2 inhibition restrains thyroid cancer growth via G1/S phase transition arrest and apoptosis mediated by DNA damage response signaling pathways
Source: Cancer Cell Int. 2022 Feb 11;22:74. doi: 10.1186/s12935-022-02496-z (PMC8840070; doi:10.1186/s12935-022-02496-z)
Supplement: Supplementary file 1 — Additional file 1: Wound-healing and transwell invasion assay. [file 12935_2022_2496_MOESM1_ESM.docx]

**Supplemental methods**

Wound-healing assay

A total of 3×10^5^ cells were seeded into 6-well plates for 24 h incubation to allow the cells reaching 90% confluence. The cells were treated with 10μM canagliflozin or dapagliflozin in 1640 medium for 24h. Cell monolayer was carefully scratched using a sterile 200μL pipette tip through the center of wells. Detached cells were removed by washing twice with 1×PBS. Cells were maintained in fresh medium for the indicated time periods. Images of the wound were recorded with a phase contrast microscope.

Transwell invasion assay

Cell invasion assay was performed using modified Boyden chambers in 24-well plates with 8 μM pore inserts (BD, Biosciences) coated with 1 mg/mL Matrigel. In the upper chamber, 5×10^4^ cells were plated in 100 μL of starving medium. The lower chamber contained 600 μL of complete medium. After 24 h of incubation at 37 °C, invaded cells were fixed with 4% paraformaldehyde and were stained with 0.5% Crystal Violet.

**Supplemental figure legends**

**Supplemental Fig.1** **Dapagliflozin inhibited TPC-1 and BCPAP cells growth.** A,B. Dapagliflozin inhibited TPC-1 and BCPAP cells viability. TPC-1 and BCPAP cells were treated with 0, 20, 40, 80μM dapagliflozin for 48 h, then cell viability were measured by CCK8. One-way ANOVA were used to determine statistical significance. C,D. Dapagliflozin inhibited TPC-1 and BCPAP cells proliferation. TPC-1 and BCPAP cells were treated with 40μM dapagliflozin, then viable cells were measured at 0, 24, 48, 72, 96h by CCK8. Repeated-measures analysis of variance were used to determine statistical significance.

**Supplemental Fig.2 SGLT2 inhibition had no effect on normal thyroid epithelial cell.** A. Canagliflozin had no effect on Nthy-ori-3-1 cells proliferation. Nthy-ori-3-1 cells were treated with 10 μM canagliflozin, then viable cells were measured at 0, 24, 48, 72, 96h by CCK8. Repeated-measures analysis of variance were used to determine statistical significance, p>0.05. B. Dapagliflozin had no effect on Nthy-ori-3-1 cells proliferation. Nthy-ori-3-1 cells were treated with 20 μM dapagliflozin, then viable cells were measured at 0, 24, 48, 72, 96h by CCK8. Repeated-measures analysis of variance were used to determine statistical significance, p>0.05. C. Canagliflozin or dapagliflozin had no effect on Nthy-ori-3-1 cells colony formation. Cells were treated with 10μM canagliflozin or 40μM dapagliflozin for 14 days, then colony formation was monitored by crystal violet stain.

**Supplemental Fig.3 The effect of SGLT2 inhibitor on thyroid cancer migration and invasion.** A. Canagliflozin had no effect on TPC-1 cells migration by the wound-healing assay. B. Canagliflozin had no effect on TPC-1 and BCPAP cells invasion. The Boyden chambers invasion assay was used. We counted the numbers of cells in light microscopy fields at ×200 magnification.
