# Supplementary figures and images for: SGLT2 inhibition restrains thyroid cancer growth via G1/S phase transition arrest and apoptosis mediated by DNA damage response signaling pathways
Source: Cancer Cell Int. 2022 Feb 11;22:74. doi: 10.1186/s12935-022-02496-z (PMC8840070; doi:10.1186/s12935-022-02496-z)

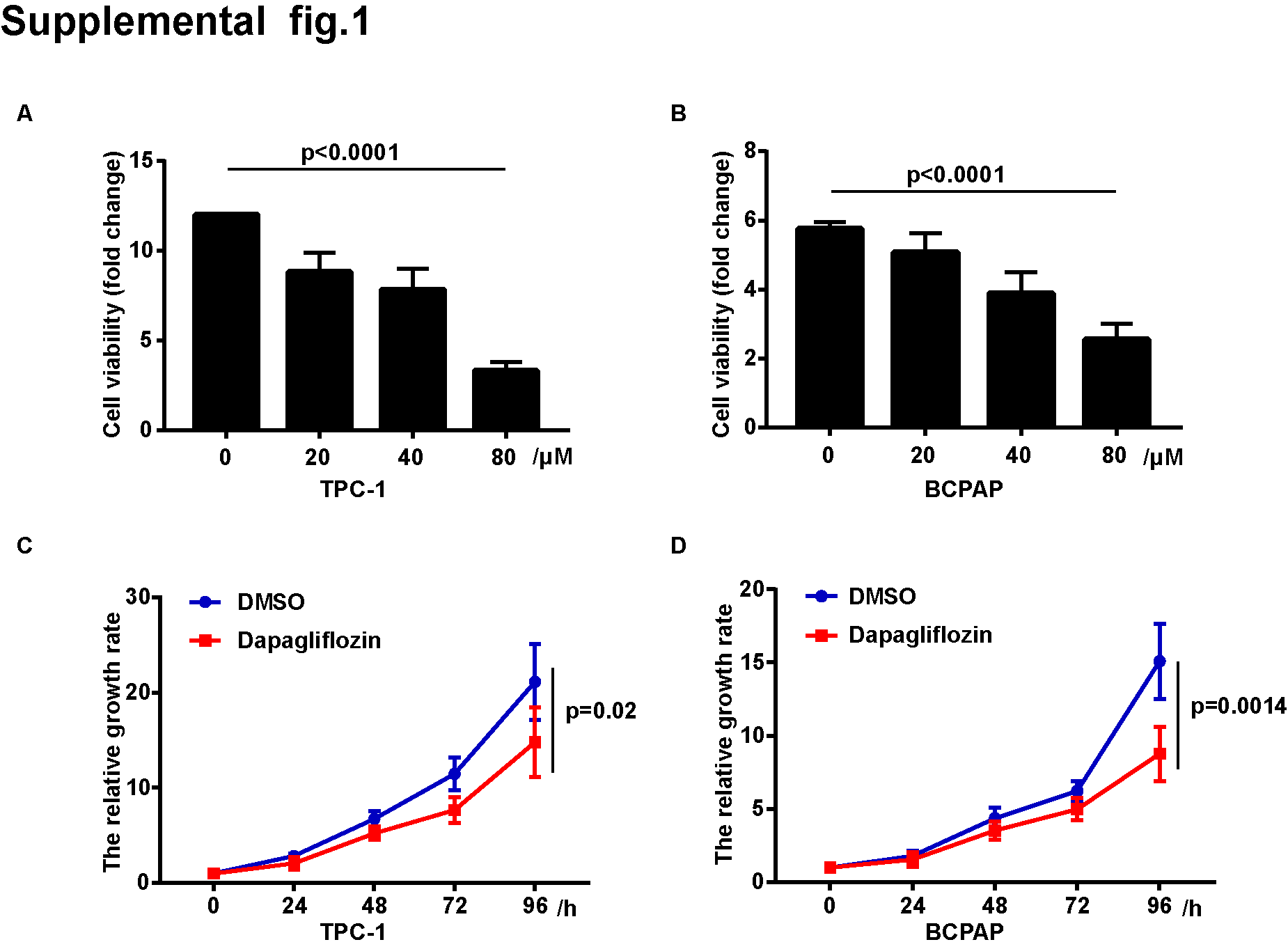

Supplement: Supplementary file 2 — Additional file 2: Figure S1. Dapagliflozin inhibited TPC-1 and BCPAP cells growth. A,B. Dapagliflozin inhibited TPC-1 and BCPAP cells viability. TPC-1 and BCPAP cells were treated with 0, 20, 40, 80μM dapagliflozin for 48 h, then cell viability were measured by CCK8. One-way ANOVA were used to determine statistical significance. C,D. Dapagliflozin inhibited TPC-1 and BCPAP cells proliferation. TPC-1 and BCPAP cells were treated with 40μM dapagliflozin, then viable cells were measured at 0, 24, 48, 72, 96h by CCK8. Repeated-measures analysis of variance were used to determine statistical significance. [file 12935_2022_2496_MOESM2_ESM.tif]

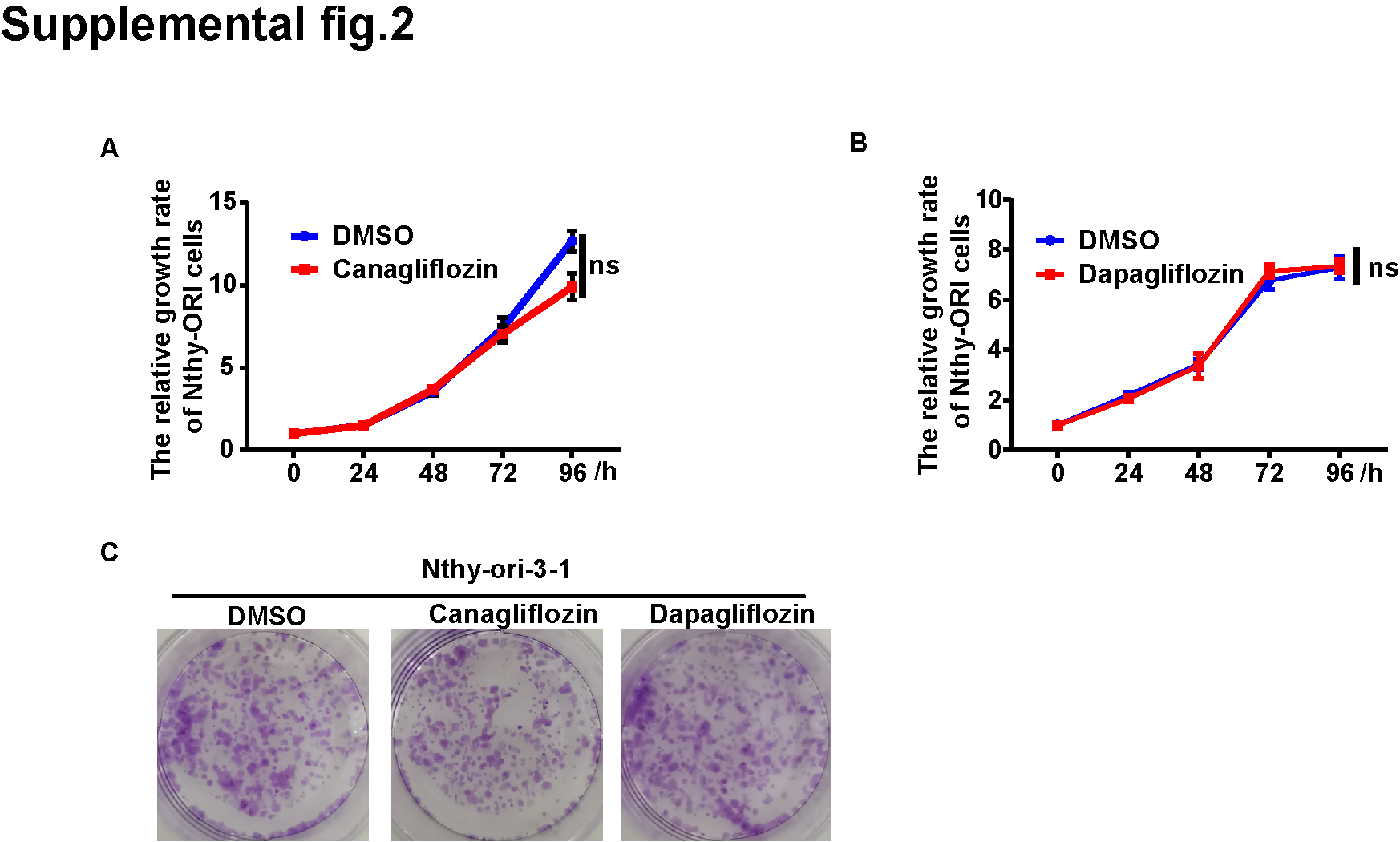

Supplement: Supplementary file 3 — Additional file 3: Figure S2. SGLT2 inhibition had no effect on normal thyroid epithelial cell. A. Canagliflozin had no effect on Nthy-ori-3-1 cells proliferation. Nthy-ori-3-1 cells were treated with 10 μM canagliflozin, then viable cells were measured at 0, 24, 48, 72, 96h by CCK8. Repeated-measures analysis of variance were used to determine statistical significance, p>0.05. B. Dapagliflozin had no effect on Nthy-ori-3-1 cells proliferation. Nthy-ori-3-1 cells were treated with 20 μM dapagliflozin, then viable cells were measured at 0, 24, 48, 72, 96h by CCK8. Repeated-measures analysis of variance were used to determine statistical significance, p>0.05. C. Canagliflozin or dapagliflozin had no effect on Nthy-ori-3-1 cells colony formation. Cells were treated with 10μM canagliflozin or 40μM dapagliflozin for 14 days, then colony formation was monitored by crystal violet stain. [file 12935_2022_2496_MOESM3_ESM.tif]

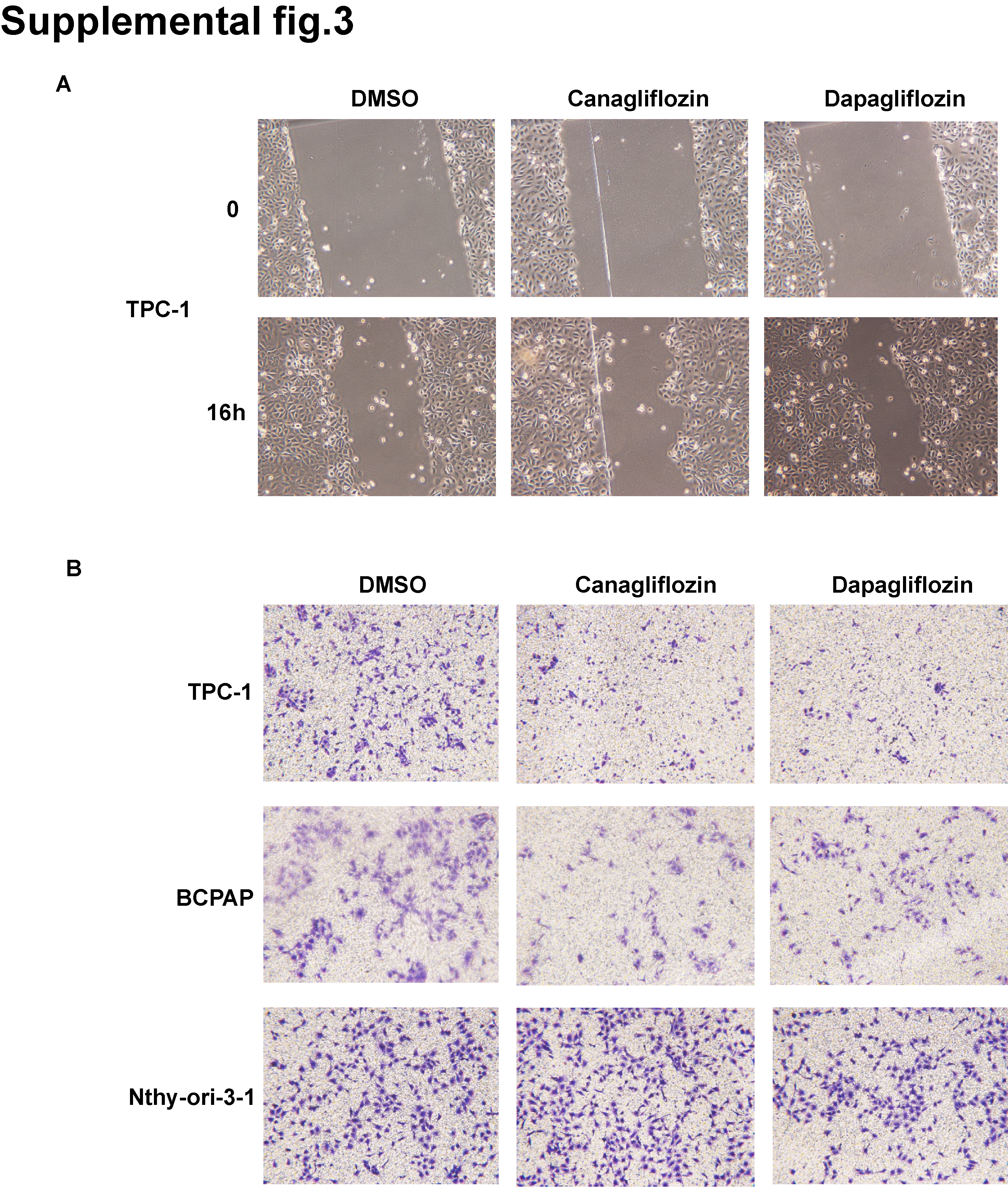

Supplement: Supplementary file 4 — Additional file 4: Figure S3. The effect of SGLT2 inhibitor on thyroid cancer migration and invasion. A. Canagliflozin had no effect on TPC-1 cells migration by the wound-healing assay. B. Canagliflozin had no effect on TPC-1 and BCPAP cells invasion. The Boyden chambers invasion assay was used. We counted the numbers of cells in light microscopy fields at ×200 magnification. [file 12935_2022_2496_MOESM4_ESM.tif]
